# Fibroblast bioenergetics to classify amyotrophic lateral sclerosis patients

**DOI:** 10.1186/s13024-017-0217-5

**Published:** 2017-10-24

**Authors:** Csaba Konrad, Hibiki Kawamata, Kirsten G. Bredvik, Andrea J. Arreguin, Steven A. Cajamarca, Jonathan C. Hupf, John M. Ravits, Timothy M. Miller, Nicholas J. Maragakis, Chadwick M. Hales, Jonathan D. Glass, Steven Gross, Hiroshi Mitsumoto, Giovanni Manfredi

**Affiliations:** 1 Feil Family Brain and Mind Research Institute, Weill Cornell Medicine, 407 East 61st Street, RR507, New York, NY 10065 USA; 2 Department of Neurology, Columbia University, New York, NY USA; 3 Department of Neuroscience, University of California San Diego, La Jolla, CA USA; 4 Department of Neurology, Washington University School of Medicine, St. Louis, MO USA; 5 Department of Neurology, Johns Hopkins University School of Medicine, Baltimore, MD USA; 6 Department of Neurology, Emory School of Medicine, Atlanta, GA USA; 7 Department of Pharmacology, Weill Cornell Medicine, New York, NY USA

**Keywords:** Bioenergetics, Mitochondria, ALS, Fibroblasts, PLS, Machine learning

## Abstract

**Background:**

The objective of this study was to investigate cellular bioenergetics in primary skin fibroblasts derived from patients with amyotrophic lateral sclerosis (ALS) and to determine if they can be used as classifiers for patient stratification.

**Methods:**

We assembled a collection of unprecedented size of fibroblasts from patients with sporadic ALS (sALS, *n* = 171), primary lateral sclerosis (PLS, *n* = 34), ALS/PLS with *C9orf72* mutations (*n* = 13), and healthy controls (*n* = 91). In search for novel ALS classifiers, we performed extensive studies of fibroblast bioenergetics, including mitochondrial membrane potential, respiration, glycolysis, and ATP content. Next, we developed a machine learning approach to determine whether fibroblast bioenergetic features could be used to stratify patients.

**Results:**

Compared to controls, sALS and PLS fibroblasts had higher average mitochondrial membrane potential, respiration, and glycolysis, suggesting that they were in a hypermetabolic state. Only membrane potential was elevated in *C9Orf72* lines. ATP steady state levels did not correlate with respiration and glycolysis in sALS and PLS lines. Based on bioenergetic profiles, a support vector machine (SVM) was trained to classify sALS and PLS with 99% specificity and 70% sensitivity.

**Conclusions:**

sALS, PLS, and *C9Orf72* fibroblasts share hypermetabolic features, while presenting differences of bioenergetics. The absence of correlation between energy metabolism activation and ATP levels in sALS and PLS fibroblasts suggests that in these cells hypermetabolism is a mechanism to adapt to energy dissipation. Results from SVM support the use of metabolic characteristics of ALS fibroblasts and multivariate analysis to develop classifiers for patient stratification.

**Electronic supplementary material:**

The online version of this article (10.1186/s13024-017-0217-5) contains supplementary material, which is available to authorized users.

## Background

Amyotrophic lateral sclerosis (ALS) is the most common form of adult onset motor neuron disease, with a yearly incidence rate of 1–2.6 cases per 100,000. ALS leads to death within 3–5 years from disease onset [1]. Typical ALS is characterized by a rapidly progressive loss of upper and lower motor neurons. However, milder forms of the disease, such as primary lateral sclerosis (PLS), cause only upper motor neuron degeneration [2]. Unfortunately, most ALS clinical trials have been unsuccessful [3], and as a result there are only two currently approved drugs for ALS, Riluzole and Edaravone, both of which only prolong life by a few months. The ineffectiveness of candidate therapies, the heterogeneity of the disease phenotype, and the diversity of ALS-linked genes support the emerging concept that distinct pathogenic mechanisms may participate in the development of ALS. For this reason, research efforts are increasingly concentrated on finding biomarkers that allow stratifying patients into groups better suited for targeted clinical trials.

Recently, a number of candidate biomarkers have been proposed, including some obtained by neuroimaging [4, 5], electrical impedance myography [6], and proteomics of cerebrospinal fluid [7–10]. However, despite their potential link to disease pathogenesis, complex cellular functions have not yet been explored as ALS biomarkers. Clearly, functional measures in living cells from affected tissues, such as the spinal cord, could be problematic, but one could envision that more accessible cell types could serve as surrogate samples. In ALS, skin fibroblasts display numerous abnormalities [11–14], many of which are shared with motor neurons [15–27]. This suggests that these apparently unaffected cells may share common pathogenic pathways with motor neurons. Furthermore, fibroblasts can be propagated in culture, frozen, and stored almost indefinitely, and transformed in cell types that are severely affected by the disease, such as motor neurons and astrocytes [28]. Furthermore, fibroblasts derived from ALS patients were used to generate a tissue-engineered skin model, which recapitulated many of the skin alterations found in ALS [29, 30]. Therefore, studying complex functional measures in fibroblasts from ALS patients could provide a promising source of new classifiers.

Here, we have investigated a large cohort of ALS fibroblasts and characterized their bioenergetic properties. We used a battery of assays to study cellular energy metabolism, and found a hypermetabolic phenotype in ALS, involving both oxidative phosphorylation and glycolysis. Importantly, using a machine learning approach on bioenergetic profiles, we provide a proof of concept that fibroblast bioenergetic markers could differentiate between ALS and PLS, and could therefore be proposed as tools for discriminating among different forms of ALS.

## Methods

### Chemicals

All chemicals used were form Sigma (St. Louis, MO), unless otherwise specified.

### Skin biopsy and fibroblast cultures

After informed consent, a punch skin biopsy was obtained from the volar part of the forearm. Skin biopsies were de-identified to protect patients’ identity. Fibroblast samples were provided to our laboratory as coded samples. Some lines were obtained from the NINDS catalog of motor neuron disease fibroblasts. Skin fibroblasts were cultured as described previously [31] in Dulbecco’s modified Eagle medium (DMEM) (Thermo Fisher Scientific, Waltham, MA) supplemented with 25 mM glucose, 4 mM glutamine, 1 mM pyruvate, and 10% fetal bovine serum (hereafter growth medium). All cultured fibroblast lines were studied at passages ranging between 5 and 10. We have not observed loss of contact inhibition in any of the lines or apparent differences in growth between any of the groups.

### Measurements of TMRM and MTG fluorescence

Skin fibroblasts were seeded at the density of 1.5 × 10^4^ cells/well in replicates of eights in 96-well tissue culture plates in growth medium and incubated at 37 °C in 5% CO_2_. The following day, cells were washed and loaded with 50 nM of the potentiometric dye Tetramethylrhodamine-methyl-ester (TMRM, 544ex, 590em; Thermo Fisher Scientific) and 450 nM MitoTracker Green (MTG, 490ex, 516em; Thermo Fisher Scientific) for 30 min at 37 °C in phenol-free DMEM containing 5 mM glucose, 4 mM glutamine, and 1 mM pyruvate. Samples were incubated in the absence or the presence of 2 μM cyanide p-trifluoromethoxyphenylhydrazone (FCCP) to completely depolarize mitochondria and obtain background TMRM and MTG fluorescence. After washing with DMEM, MTG and TMRM fluorescence were simultaneously recorded in a plate reader equipped with a polychromator (Spectramax M5; Molecular Devices Sunnyvale, CA). Background fluorescence was subtracted from the total fluorescence. MTG and TMRM fluorescence values were expressed as relative fluorescence units per milligram of total cellular proteins measured with the DC Protein Assay (BioRad, Hercules, CA).

### Measurement of ATP content

Fibroblasts were seeded at the density of 1.5 × 10^4^ cells/well in replicates of nines in 96-well tissue culture plates in growth medium incubated at 37 °C in 5% CO_2_. The next day cells were incubated in triplicates in DMEM containing 5 mM glucose, 4 mM glutamine, and 1 mM pyruvate (ATP baseline), or DMEM containing 4 mM glutamine, 1 mM pyruvate, and 5 mM 2-deoxy-D-glucose (2DG) to bock glycolysis (ATP 2DG), or DMEM containing 5 mM glucose, 4 mM glutamine, 1 mM pyruvate, and 1 μM oligomycin to block the mitochondrial ATPase (ATP Oligo). After 90 min incubation, cells were washed with phosphate buffered saline (PBS) and lysed in 30 μl tichloroacetic acid (2.5% *W*/*V*) on ice for 30 min. Following lysis, 20 μl aliquots were transferred into a separate plate for protein determination (DC Protein Assay). 45 μl Tris-acetate buffer (400 mM, pH = 8.0) was added to the remaining lysate. Cellular ATP content was measured after addition of 20 μl of luciferase reagent (Promega, Madison, WI) in a luminescence plate reader (Spectramax M5). Luminescence values were normalized against an ATP standard.

### Measurements of oxygen consumption and extracellular acidification

Oxygen consumption rate (OCR) and extracellular acidification rate (ECAR) were measured with a XF96 Extracellular Flux Analyzer (Agilent, Santa Clara, CA). Cell lines were seeded in 12 wells of a XF 96-well cell culture microplate (Agilent) at a density of 1 × 10^4^ cells/well (cells reach confluency on the experimental day) in 200 μL of growth medium and incubated for 24 h at 37 °C in 5% CO_2_. After replacing the growth medium with 200 μL of XF Assay Medium (Agilent) supplemented with 5 mM glucose, 1 mM pyruvate and 4 mM glutamine, pre-warmed at 37 °C, cells were degassed for 1 h before starting the assay procedure, in a non-CO_2_ incubator. OCR and ECAR were recorded at baseline followed by sequential additions of 1 μM oligomycin, 2 μM FCCP and 0.5 μM Antimycin A plus 0.5 μM Rotenone. Non-mitochondrial oxygen consumption (in the presence of AA + Rot) was subtracted from all OCR values and technical replicates outside of two standard deviations of the means were discarded for both ECAR and OCR. Values were normalized by the mean protein value of each line. The measurement of OCR and ECAR in galactose medium was performed as described above, with the exception that the growth medium and assay medium contained no glucose and was supplemented with 5 mM galactose.

### Lactate excretion rate measurement

Lactate production was measured using a kit (EnzyFluoTM L-lactate Assay Kit (EFLLC-100), BioAssay Systems) based on a fluorescent probe linked to NADH generated from lactate. Cell lines were seeded in XF 96-well cell culture microplates as described for OCR and ECAR measurements. On the experimental day, the growth medium was replaced with 200 μL of XF Assay Medium supplemented with 5 mM glucose, 1 mM pyruvate and 4 mM glutamine, pre-warmed at 37 °C. Cells were allowed to excrete lactate by incubation for 210 min at 37 °C in 5% CO_2_. Aliquots of the medium were then collected, and diluted 5 fold for the assay. Lactate standards diluted in Assay medium were used for quantification.

### Statistical analyses

Bioenergetic features were tested for normality by D’Agostino-Pearson test (scipy v0.15.1; www.scipy.org), which combines skewness and kurtosis to produce an omnibus test of normality. Since none of the parameters passed the normality test, we used non-parametric tests. Differences amongst groups were compared using Kruskal–Wallis one-way ANOVA, followed by Dunn’s multiple comparison test, as post hoc analysis (scipy v0.15.1). Correlations amongst bioenergetic features were tested using Spearman rank-order correlation coefficient. The *p*-values of correlations were adjusted for multiple comparisons by Benjamini-Hochberg correction with a false discovery rate set to <0.05. Data in the text are presented as % average (±95% confidence intervals of the differences). There were no correlations between any of the measured bioenergetics parameters and patient age, sex, or cell line passage number. Therefore, no adjustment for these parameters was necessary.

### Support vector machines

The complexity and performance of the SVM model is controlled by tunable parameters (class weights, kernels, penalties and gamma values). For each classification problem (i.e., control vs. disease, sALS vs. PLS), we tested an array of 1120 different sets of model parameters (grid search). A less complex model would have lower performance, but a more complex one would “overfit” the data, and the resulting decision boundary would follow the noise of the samples rather than inherent patterns that generalize well to the population. To find the best performing SVM that does not overfit, we used the well-established metod of k-fold cross-validation as a measure model performance: first the data was randomly divided into 10 sets, then for each set of model parameters the SVM was trained on 9 and validated on one set of the data. This step was repeated 10 times, using a different set as the validation set each time. Model performance was calculated as the average of the 10 validation performances (% accuracy). Fitting SVMs and generating receiver operating characteristic (ROC) curves were performed using scikit-learn v0.18 (www.scikit-learn.org). ROC curves are the averages of the 10 cross-validation sets.

## Results

### Clinical features of study subjects

Table 1 summarizes the clinical characteristics of the sALS, PLS, *C9Orf72*, and control subjects whose de-identified fibroblasts were utilized for this study. sALS patients (*n* = 171) were clinically defined based on definite or probable ALS diagnosis. sALS patients did not have family history of ALS are were negative for SOD1 and *C9orf72* mutations*.* PLS patients (*n* = 34) were clinically defined on the basis of pure upper motor neuron disease, >5 years after symptom onset, normal electromyogram, and no definable causes. As expected, there were more males in both the sALS and PLS groups, consistent with higher frequency of the disease in males [32]. Also predictably, the rate of progression (i.e., the rate at which the ALS Functional Rating Scale, ALSFRS, worsens) and the forced vital capacity (FVC) decline were significantly less severe in the PLS group than in sALS (mean: 17.7%, CI: 7.2 to 27.7%, *p* = 1.9E-18 and mean: 117.8%, CI: 107.1 to 128.1%, *p* = 2.9E-4 respectively), consistent with the milder phenotype in PLS. The age of disease onset was significantly earlier in the PLS group (mean: 89.6%, CI: 84.2 to 95.0%, *p* = 6.1E-05). We included in the study fibroblasts from patients with *C9orf72* expansion who had ALS (*n* = 12) or PLS (n = 1). However, we did not compare the clinical features of the genetically defined *C9orf72* group, because of the relatively low number of samples available.

**Table 1:** Clinical characteristics of study subjects

	n	Sex F/M	Age at onset	Age at biopsy	ALSFRS	Rate of progression	FVC (%)	BMI	Onset S/B
Controls	91	0.88	–	60.3 (47–83)	–	–	–	NA	–
sALS	171	0.67	58.3 (26–79)	59.7 (27–80)	34.2 (8–47)	1.0 (0.06–3.4)	76.1 (6–138)	26.4 (16.2–39.7)	1.98
PLS	34	0.79	51.7* (32–74)	59.2 (41–81)	33.2 (14–44)	0.2* (0.07–0.37)	89.6* (31–143)	26.9 (19–34.6)	2.67
*C9Orf72*	13	1.50	56.3 (40–70)	58.3 (38–72)	34.9 (30–41)	0.9 (0.24–2.0)	79.3 (38–115)	27.8 (20.5–50.4)	3.00

Values indicate averages and values in brackets indicate ranges. Sex F/M, is the female to male ratio; ALSFRS, is the ALS functional rating scale at time of skin biopsy; Rate of progression is the % of ALSFRS decline per month; FVC is the forced vital capacity at time of skin biopsy expressed as % of normal; BMI is the body mass index at time of skin biopsy; Onset S/B is the ratio of site of disease onset, spinal (S) or bulbar (B). *NA* not available

*p < 0.005 PLS vs. ALS, based on Mann-Whitney U test

### Bioenergetic characterization of sALS, PLS, and C9Orf72 fibroblasts

**Fig. 1:**
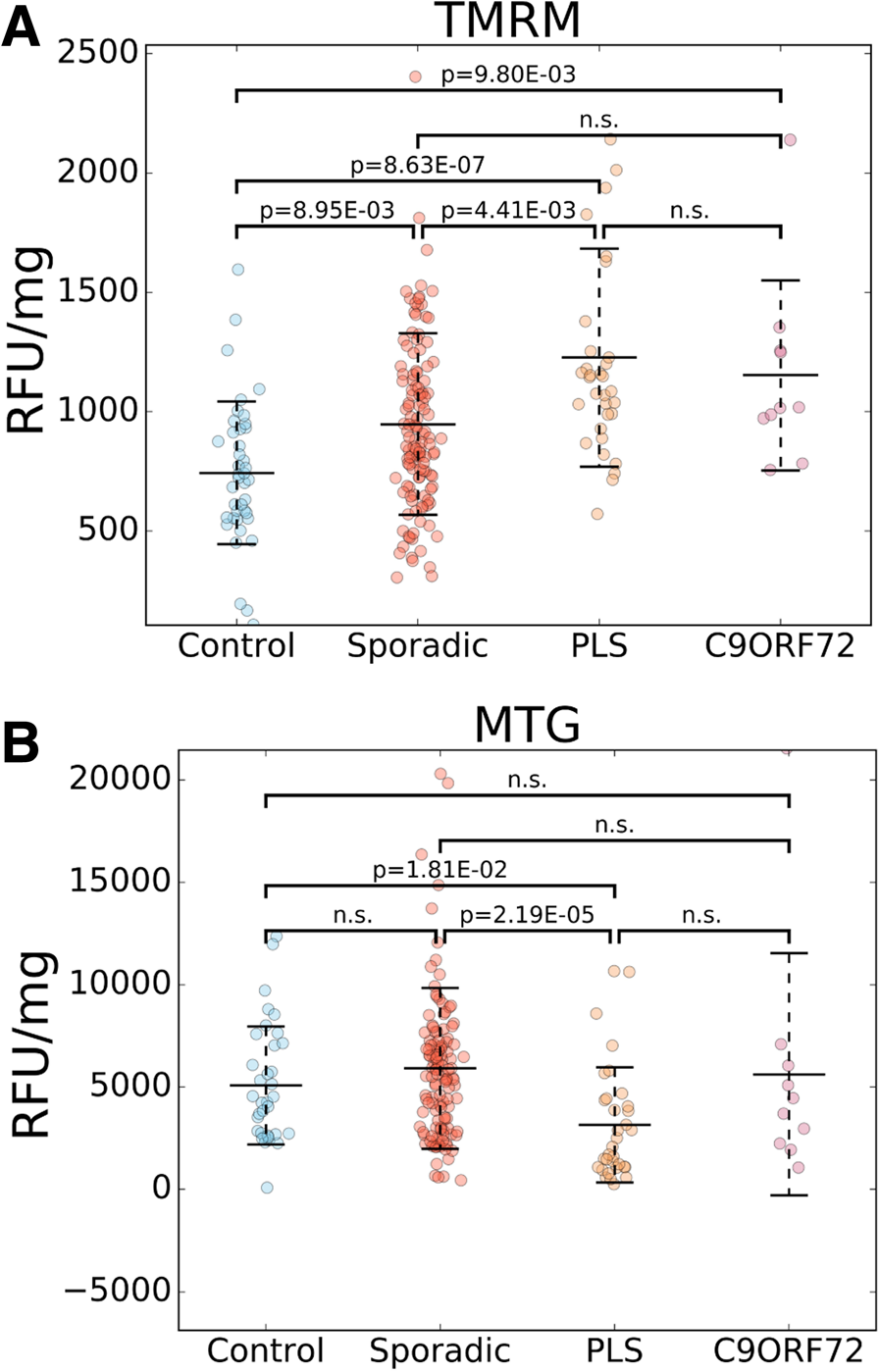
Higher mitochondrial membrane potential in fibroblasts from patients with motor neuron disease. Scatter plots of TMRM **a** Control mean: 743.4, SD: 298.5; sALS mean: 948.3, SD: 380.3, PLS mean: 1227.3, SD: 456.7, *C9Orf72* mean: 1153.0, SD: 397.5) and MTG **b** Control mean: 5080.9, SD: 2886.7, sALS mean: 5918.7, SD: 3939.6, PLS mean: 3153.6, SD: 2813.2; *C9Orf72* mean: 5616.9, SD: 5911.8) values in sALS, PLS, *C9Orf72*, and control fibroblast lines. Middle bars represent the average values and error bars show standard deviations. RFU: relative fluorescence units. *p*-values are indicated where there was a significant difference between two groups. n.s.: no significant difference. *n* = 127 sALS, *n* = 33 PLS, *n* = 10 *C9Orf72, n* = 41 controls

To generate a comprehensive bioenergetic profile of fibroblast lines in disease and control groups we measured the following parameters: mitochondrial membrane potential, cellular ATP content, cell respiration, and glycolysis. The fluorescence intensity of tetramethylrhodamine methyl ester (TMRM), an indicator of mitochondrial membrane potential, was significantly higher in all disease groups relative to controls (sALS mean: 127.6%, CI: 112.5 to 142.8%; PLS mean: 165.1%, CI: 142.1 to 189.3%; *C9Orf72* mean: 155.1%, CI: 125.5 to 191.5%, Fig. 1a). To determine whether increased mitochondrial membrane potential could be attributed to differences in mitochondrial content, we measured the fluorescence of MitoTrackerGreen (MTG), a dye that is trapped and enriched in mitochondria, with minimal dependence on membrane potential [31], and can therefore be used as a readout of mitochondrial content. sALS and *C9Orf72* lines showed no differences in MTG fluorescence relative to controls (Fig. 1b), whereas PLS had a significant decrease in MTG fluorescence (mean: 62.1%, CI: 36.3 to 88.8%). Since increased TMRM fluorescence in sALS and PLS was not matched by proportional increases in MTG fluorescence, we inferred that higher TMRM fluorescence was attributable to increased mitochondrial membrane potential, and not to mitochondrial content. Note that the number of samples tested for TMRM and MTG fluorescence was smaller than the total number of sALS lines available, because a subset of them were assessed for these two bioenergetic parameters in a previous study, which also indicated higher TMRM values in a smaller cohort of sALS and PLS lines [31].

**Fig. 2:**
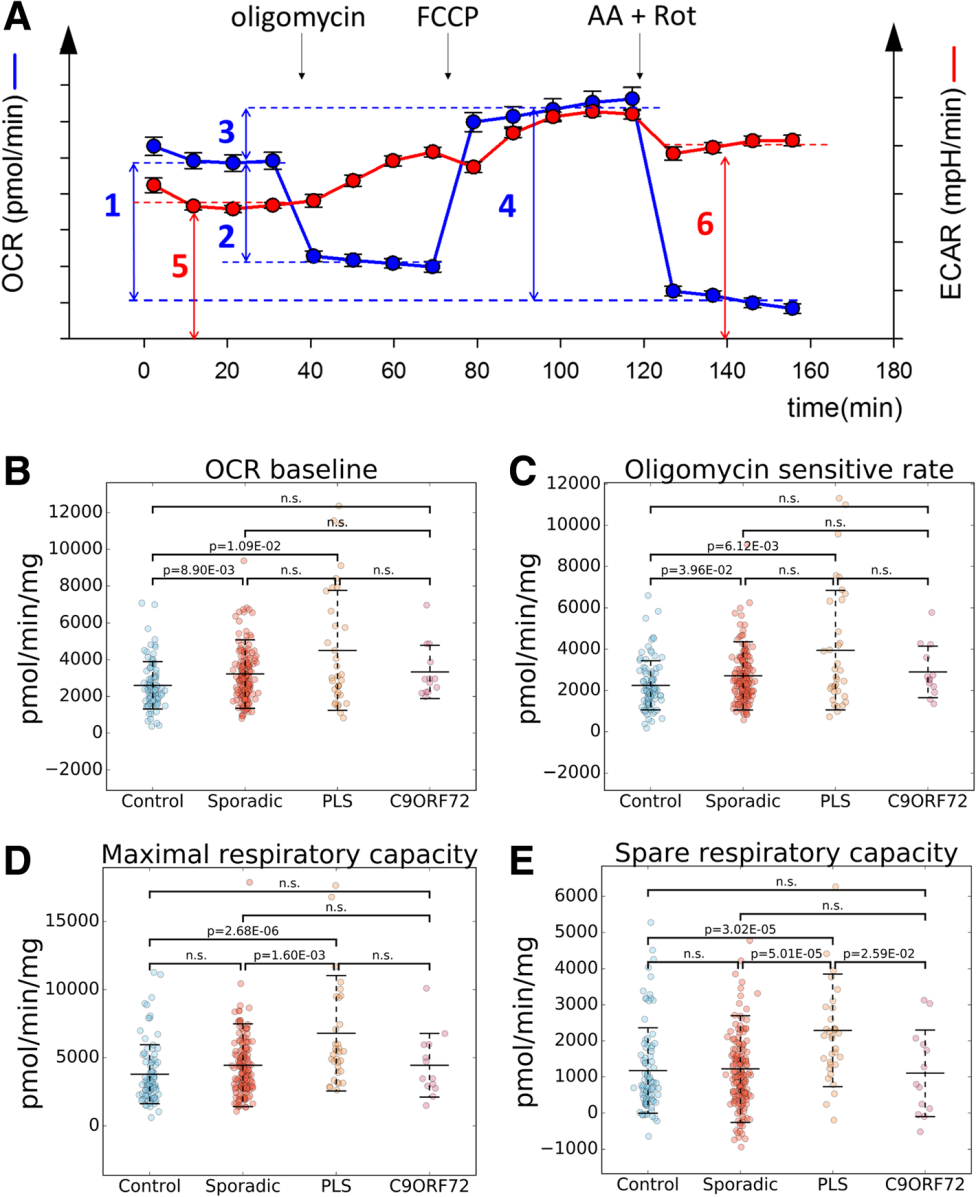
Higher oxygen consumption rates in sALS and PLS fibroblasts. **a** Schematic illustration of a typical flux experiment and the calculated metrics (1: OCR baseline, 2: oligomycin sensitive rate, 3: spare respiratory capacity, 4: maximal respiratory capacity, 5: ECAR baseline, 6: ECAR AA-Rot). B-E: scatter plots of OCR baseline **b** Control mean: 2606.9, SD: 1290.5; sALS mean: 3224.0, SD: 1864.5; PLS mean: 4504.6, SD: 3259.3; *C9Orf72* mean: 3338.2, SD: 1450.7), oligomycin sensitive rate **c** Control mean: 2248.2, SD: 1193.9; sALS mean: 2707.6, SD: 1656.9; PLS mean: 3947.9, SD: 2892.3; *C9Orf72* mean: 2900.4, SD: 1251.4), maximal respiratory capacity **d** Control mean: 3784.6, SD: 2166.9; sALS mean: 4445.7, SD: 3048.2; PLS mean: 6797.1, SD: 4247.2; *C9Orf72* mean: 4443.5, SD: 2339.5), spare respiratory capacity **e** Control mean: 1177.7, SD: 1187.2; sALS mean: 1221.7, SD: 1476.5; PLS mean: 2292.5, SD: 1563.6; *C9Orf72* mean: 1105.3, SD: 1195.8). Values are shown comparing sALS, PLS, *C9Orf72*, and control lines. Middle bars represent the average values and error bars show standard deviations. *p*-values are indicated where there was a significant difference between two groups. n.s.: no significant difference. *n* = 171 sALS; *n* = 34 PLS, *n* = 13 *C9Orf72*, *n* = 91 controls

Next, we measured mitochondrial OCR (Fig. 2a, blue curve) using flux analysis. In this experiment, baseline OCR is first measured, followed by the addition of the ATPase inhibitor oligomycin, which decreases OCR, as the proton motive force cannot be used for ATP production. The oligomycin sensitive OCR is calculated by subtracting oligomycin OCR rate from baseline. Then, the proton motive force is dissipated using the uncoupler FCCP, which allows the respiratory chain to consume oxygen at its fastest rate. The FCCP OCR rate is considered as maximal respiratory capacity, and the spare respiratory capacity is calculated as the difference between maximal and baseline OCR. Lastly, a mixture of the respiratory chain complex III and I inhibitors (Antimycin A and Rotenone, AA + Rot, respectively) are added to assess non-mitochondrial respiration, which is considered as background.

Relative to controls, sALS and PLS had elevated baseline OCR (sALS mean: 123.7%, CI: 109.1 to 138.9%; PLS mean: 172.8%, CI: 131.6 to 216.4%, Fig. 2b), oligomycin sensitive OCR (sALS mean: 120.4%, CI: 105.3 to 136.0%; PLS mean: 175.6%, CI: 134.5 to 220.5%, Fig. 2c), and maximal respiratory capacity (sALS mean: 117.5%, CI: 100.8 to 135.0%; PLS mean: 179.6%, CI: 142.0 to 220.4%, Fig. 2d). Spare respiratory capacity was only significantly elevated in PLS lines (mean: 194.7%, CI: 149.4 to 243.7%, Fig. 2e). Comparing sALS, PLS and *C9Orf72*, there was no difference in baseline OCR and oligomycin sensitive respiration, but relative to PLS sALS had lower maximal capacity (mean: 65.4%, CI: 43.0 to 85.8%), and both sALS and *C9Orf72* had lower spare respiratory capacity (sALS: mean: 53.3%, CI: 27.7 to 76.9%; C9Orf72: mean: 48.2%, CI: 12.9 to 83.3% Fig. 2e).

**Fig. 3:**
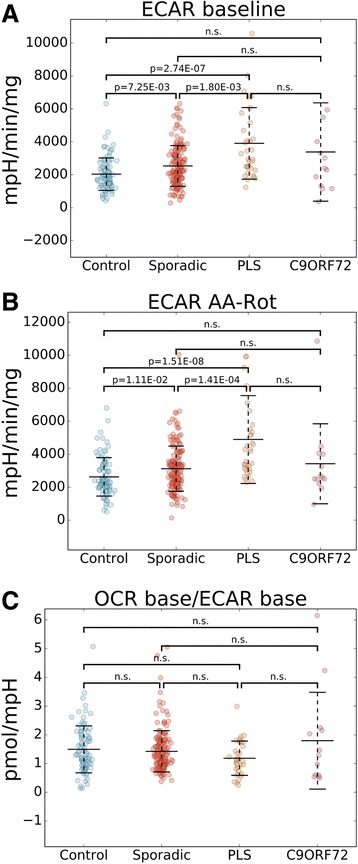
Higher extracellular acidification rates in sALS and PLS fibroblasts. Scatter plots of ECAR baseline **a** Control mean: 2035.9, SD: 987.4; sALS mean: 2536.0, SD: 1237.8; PLS mean: 3909.1, SD: 2175.4; *C9Orf72* mean: 3381.9, SD: 2988.0), ECAR AA-Rot **b** Control mean: 2631.1, SD: 1164.4; sALS mean: 3126.2, SD: 1366.6; PLS mean: 4895.2, SD: 2661.5; *C9Orf72* mean: 3426.1, SD: 2425.9), and OCR base/ECAR base **c** Control mean: 1.5, SD: 0.8; sALS mean: 1.4, SD: 0.7; PLS mean: 1.2, SD: 0.6; *C9Orf72* mean: 1.8, SD: 1.7) values are shown comparing sALS, PLS, *C9Orf72*, and control lines. The method by which features were calculated is illustrated in Fig. 2a. Middle bars represent the average values and error bars show standard deviations. p-values are indicated where there was a significant difference between two groups. n.s.: no significant difference. n = 171 sALS; n = 34 PLS, n = 13 *C9Orf72*, n = 91 controls

In parallel to OCR, the flux analyzer allows for measurement of ECAR (Fig. 2a, red curve). Relative to controls, both sALS and PLS cells had higher baseline ECAR (sALS: mean: 124.6%, CI: 111.0 to 138.0%; PLS: mean: 192.0%, CI: 157.4 to 230.5%, Fig. 3a). ECAR AA + Rot (i.e., maximal ECAR when the respiratory chain is fully inhibited) was also higher (sALS: mean: 118.8%, CI: 17.0 to 130.5%; PLS: mean: 186.1%, CI: 153.5 to 222.9%, Fig. 3b). Moreover, PLS had higher ECAR baseline (mean: 154.1%, CI: 126.5 to 185.1%, Fig. 3a) and ECAR AA + Rot (mean: 156.6%, CI: 129.9 to 187.3%, Fig. 3b) than sALS.

Taken together, these results showed that sALS and PLS fibroblasts upregulate both oxidative phosphorylation and glycolysis. Furthermore, the extent of relative OCR and ECAR increase was similar in the two groups, suggesting that both bioenergetic pathways are similarly upregulated. This was confirmed by calculating the ratio between OCR baseline and ECAR baseline, which was not different among the groups (Fig. 3c), indicating that there was no metabolic shift towards either glycolysis or oxidative phosphorylation. Of note, in highly aerobic states, CO_2_ production from mitochondria can contribute to ECAR. However, it was empirically determined that in cells with low baseline OCR/ECAR ratio (e.g., < 4) CO_2_ has a negligible contribution to ECAR [33]. Since in fibroblasts the average OCR/ECAR ratio was approximately 1.5, the CO_2_ contribution to ECAR was likely not significant, suggesting that glycolysis was the major contributor to ECAR. Furthermore, since under conditions of full respiratory chain inhibition CO_2_ production in the Krebs cycle is strongly attenuated, the interpretation of higher glycolysis is also supported by the higher ECAR values in sALS and PLS after AA + Rot addition. To test experimentally the assumption that ECAR reflects lactate excretion we measured lactate production rates in a subset of 12 sALS, 12 PLS, and 12 control lines, under baseline conditions. Lactate production rate and ECAR baseline were significantly correlated (*p* = 0.02, data not shown), indicating that medium acidification reflects lactate excretion. Collectively, these results suggest that sALS and PLS fibroblasts have a hypermetabolic phenotype involving both oxidative phosphorylation and anaerobic glycolysis.

**Fig. 4:**
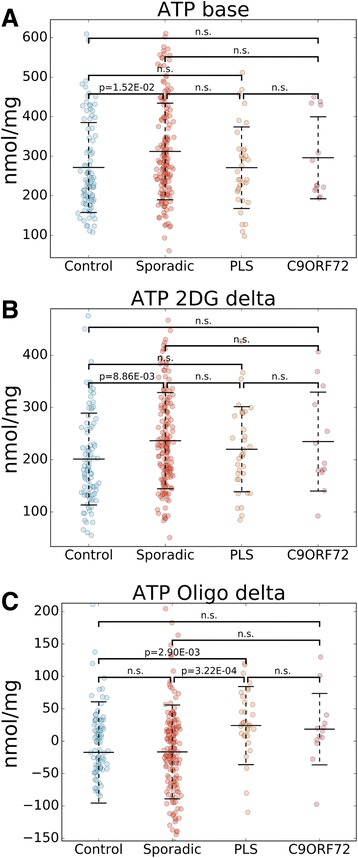
ATP content in sALS and PLS fibroblasts. Scatter plots of baseline ATP content **a** Control mean: 271.6, SD: 113.7; sALS mean: 312.1, SD: 122.2; PLS mean: 271.1, SD: 103.2; *C9Orf72* mean: 296.1, SD: 103.8), ATP content lost after 2DG treatment **b** Control mean: 201.2, SD: 87.8; sALS mean: 236.4, SD: 92.1; PLS mean: 220.1, SD: 81.4; *C9Orf72* mean: 234.7, SD: 94.6), and ATP content lost after oligomycin treatment **c** Control mean: -17.3, SD: 78.1; sALS mean: -16.6, SD: 72.4; PLS mean: 24.1, SD: 60.2; *C9Orf72* mean: 18.6, SD: 55.1) are shown comparing sALS, PLS, *C9Orf72*, and control lines. Groups were compared using Kruskal–Wallis one-way analysis of variance followed by Dunn’s post hoc analysis. Middle bars represent the average values and error bars show standard deviations. *p*-values are indicated where there was a significant difference between two groups. n.s.: no significant difference. n = 171 sALS; n = 34 PLS, n = 13 *C9Orf72*, n = 91 controls

Next, we measured cellular steady-state ATP levels at baseline and after 90 min treatment with either 2DG to inhibit glycolysis or oligomycin to inhibit mitochondrial ATP production. In sALS, we found higher baseline ATP content compared to controls (mean: 114.9%, CI: 13.6 to 125.8%, Fig. 4a). Interestingly, PLS fibroblasts did not show higher ATP content, despite having the highest average respiration and glycolytic fluxes among all groups. The decline in ATP content after 2DG (ATP 2DG delta) was greater in sALS relative to controls (mean: 117.5%, CI: 106.4 to 128.7%, Fig. 4b). However, neither PLS nor *C9Orf72* lines had ATP 2DG delta greater than controls. When oxidative phosphorylation was inhibited (ATP Oligo delta), we observed no significant decline in ATP content in controls or sALS, while PLS showed a small but significant decline (−8.9%, Fig. 4c). Overall, these results suggest that sALS lines are more dependent on glucose utilization for ATP maintenance than controls, PLS or *C9Orf72*. Additionally, PLS is the only group that exhibits dependency on oxidative phosphorylation for ATP maintenance.

We performed correlation analyses of bioenergetic parameters of fibroblasts to assess if the interdependence of the parameters differed among groups (Table 2). The underlying assumption was that parameters would correlate when they were co-regulated. First, these analyses confirmed that all groups were dependent on glycolysis for energy production, as baseline ATP and sensitivity to 2DG (ATP 2DG delta) were strongly correlated. Second, the maximal glycolytic rate (ECAR AA-Rot) significantly correlated with the maximal OCR rate (OCR Max) in both sALS and PLS. Similarly, there was a direct correlation between OCR baseline and ECAR baseline in sALS and PLS, but not in controls. Taken together, these correlations suggest that in sALS and PLS lines glycolytic and oxidative fluxes are co-regulated. Despite these similarities, there were also differences between sALS and PLS. For example, only in PLS there was a negative correlation between the OCR/ECAR ratio and ATP content. Furthermore, only PLS cells showed a negative correlation between OCR/ECAR ratio and ATP 2DG delta.

**Table 2:** Correlations among bioenergetics features

Feature 1	Feature 2	Control		sALS		PLS		*C9Orf72*	
		R	p value	R	*p* value	R	p value	R	p value
ATP baseline	ATP 2DG delta	0.922	6.45E-37	0.924	7.07E-71	0.941	4.61E-15	0.648	n.s.
ECAR AA-Rot	OCR Max	0.638	9.62E-11	0.683	6.00E-24	0.826	2.07E-08	0.489	n.s.
ECAR baseline	OCR baseline	0.234	n.s.	0.574	1.49E-15	0.551	3.01E-03	0.170	n.s.
OCR base/ECAR base	ATP baseline	0.211	n.s.	−0.017	n.s.	−0.508	7.45E-03	0.384	n.s.
OCR base/ECAR base	ATP 2DG delta	0.248	n.s.	0.012	n.s.	−0.491	9.53E-03	0.264	n.s.
OCR baseline	ATP baseline	0.182	n.s.	0.155	n.s.	−0.434	2.42E-02	0.445	n.s.
ECAR baseline	ATP baseline	0.012	n.s.	0.124	n.s.	0.005	n.s.	−0.148	n.s.

Values indicate Spearman’s correlation coefficients (R) and p-values (corrected by the Benjamini-Hochberg method with a false discovery rate set to <0.05). n.s., not significant

To test the ability of fibroblasts to respond to forced oxidative metabolism we grew a subset of lines in medium containing galactose instead of glucose for 24 h. In these conditions, fibroblasts are forced to oxidize glycolysis-derived pyruvate for energy production, because galactose is not converted to glucose-6P as efficiently as glucose [34]. We tested the 12 controls, 12 sALS, and 12 PLS lines that had ECAR and OCR values closest to the average of their respective groups. As expected, in galactose medium baseline OCR was faster than in glucose medium (compare Additional file 1: Figure S1A and Figure 2B), while ECAR was lower (compare Additional file 1: Figure S1E and Figure 3A). This was also apparent from the increase of the OCR/ECAR ratio from approximately 1.5 in glucose to 3.5 in galactose (compare Additional file 1: Figure S1F and Figure 3C). The differences in baseline OCR and ECAR between sALS and controls, PLS and controls, and sALS and PLS that was observed in glucose was not detected in galactose (Additional file 1: Figure S1A and S1E). These results suggest that control cells can upregulate OCR in galactose to match sALS and PLS. Interestingly, in galactose, the spare respiratory capacity was significantly lower in sALS than controls (Additional file 1: Figure D), while in PLS it was similar to controls. Since in glucose the spare respiratory capacity was higher in sALS and PLS than control (Fig. 2e), we interpret the result in galactose as an indication that ALS and PLS fibroblasts have respiration closer to maximal in glucose and cannot upregulate it much more when placed in galactose.

### Stratification of ALS patients based on individual bioenergetic features

We separated our cohort of sALS fibroblast in two equally sized groups by the median values of key bioenergetic parameters: TMRM fluorescence (mitochondrial membrane potential), ECAR AA + Rot (maximal glycolytic activity), and oligomycin sensitive OCR (ATP synthesizing respiration). We then compared the two groups (i.e., with above median and below median values) for key clinical parameters: sex, age of disease onset, site of onset (i.e., bulbar vs. spinal), rate of disease progression, FVC at time of biopsy. We found that patients with high ECAR AA + Rot had significantly higher FVC (14%, *p* = 0.01) and more frequent spinal onset (25%, *p* = 0.02). We also found that patients with high TMRM had a faster rate of decline (18%, *p* = 0.04). Similar analyses were not performed for PLS or *C9Orf72* lines, because the number of samples was too small to obtain adequately sized groups. Indeed, although interesting, the significant differences between upper and lower halves of the sALS lines were not large. Furthermore, significant liner correlations between individual key bioenergetic and clinical parameters, after correction for multiple correlations, were not found (not shown). Therefore, based on the available samples, we suggest that individual bioenergetic parameters in fibroblast lines may not be adequate to provide definite clinical classifications.

### Supervised machine learning on bioenergetic profiles classifies sALS and PLS fibroblasts with high specificity

As no individual bioenergetic measure had sufficient sensitivity or specificity to be used as a tool for classification by itself (data not shown), we took advantage of the high dimensionality of the data gathered and performed multivariate analysis. The following 12 features from 301 records (control, sALS, PLS, and *C9Orf72* combined) were used: TMRM, MTG, ECAR base, ECAR AA-Rot, OCR baseline, oligomycin sensitive respiration, spare respiratory capacity, maximal respiration, OCR baseline/ECAR baseline, ATP baseline, ATP 2DG delta and ATP Oligo delta. The goal was to determine if fibroblast groups could be clustered and predicted based purely on their bioenergetic features. Importantly, considering that such tool could have translational applications in helping to stratify patients, we wanted to establish proof of principle that multivariate analyses could distinguish between sALS and PLS. To this end, we utilized support vector machines (SVM), which are trained to fit non-linear decision boundaries to high dimensional data.

**Fig. 5:**
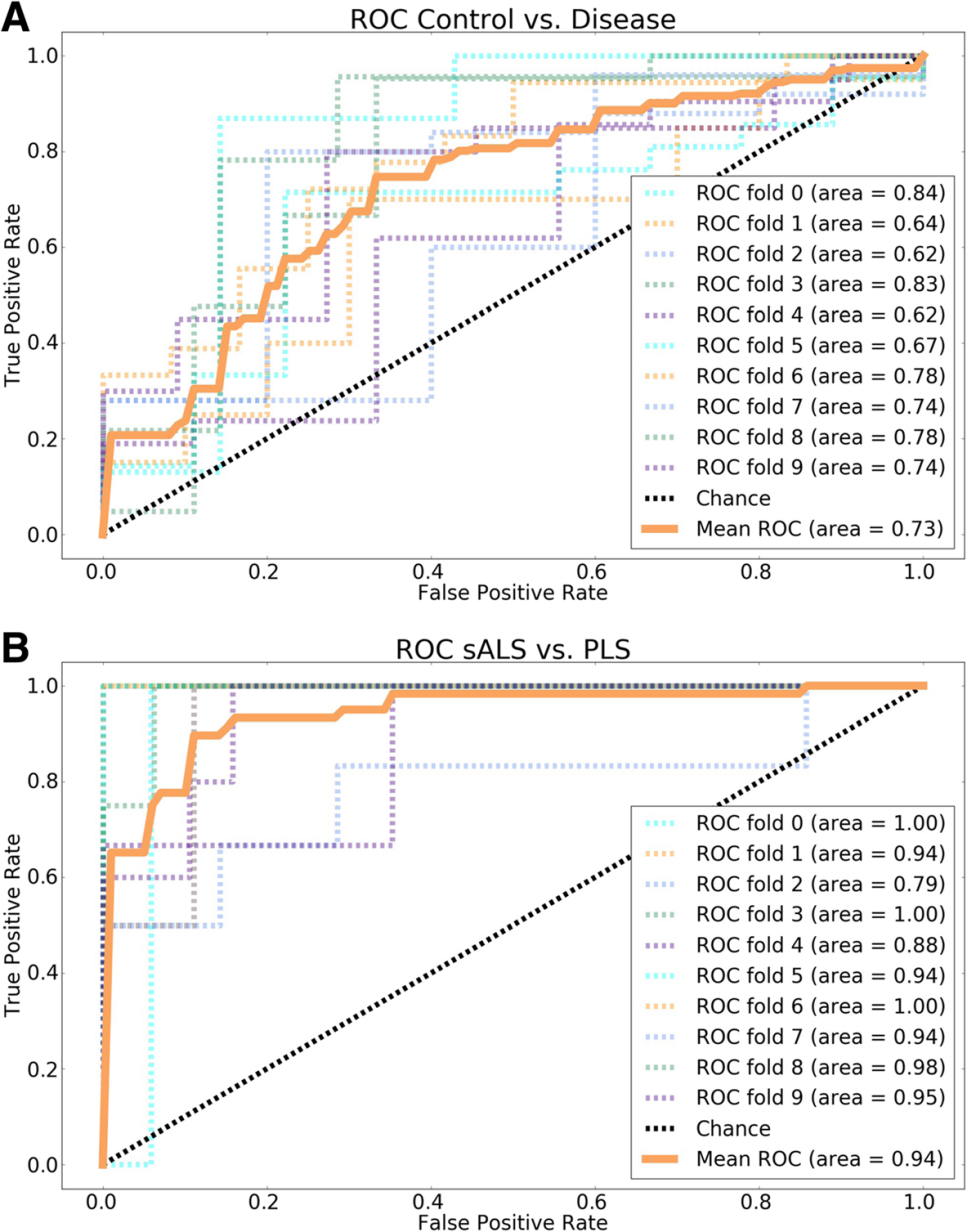
Receiver operating characteristic (ROC) curves of SVM classifiers distinguishes lines with motor neuron disease from controls (**a**), and sALS from PLS (**b**). SVMs were trained to distinguish between two groups, based on 12 bioenergetics features. Each continuous orange ROC curve represents the mean of 10 cross-validation curves, each shown as a dotted line. Values of the area under the curve for each ROC curve (0–9 fold ROC and mean ROC) are indicated in the boxes

First we sought to classify control fibroblasts versus all disease groups combined (i.e., sALS, PLS and *C9Orf72*). Receiver operating characteristics (ROC) curves were generated based on the SVM classifier that yielded the highest accuracy (probability of correct assignment of samples to their respective groups). The best performing SVM yielded good sensitivity (88.5%, CI: 84.2 to 92.7%), but low specificity (38.1%, CI: 27.7 to 48.5%) (Fig. 5a). Other parameters of the performance of this SVM classifier included, positive predictive value (78.7%, CI: 73.6 to 83.8%), negative predictive value (56.1%, CI: 43.3 to 69.0%), false positive rate (61.9%, CI: 51.5 to 72.3%), false negative rate (11.5%, CI: 7.3 to 15.8%), and false discovery rate (21.3%, CI: 16.2 to 26.4%). Interestingly, our best performing SVM to classify sALS versus PLS (Fig. 5b) yielded good sensitivity (70.6%, CI: 55.3 to 85.9%), and high specificity (98.8%, CI: 97.2 to 100.0%), with a positive predictive value of 92.3%, CI: 82.1 to 100.0%, negative predictive value of 94.4%, CI: 91.0 to 97.8%, false positive rate of 1.2%, CI: 0.0 to 2.8%, false negative rate of 29.4%, CI: 14.1 to 44.7%, and false discovery rate of 7.7%, CI: 0.0 to 17.9%. In summary, the SVM analysis of fibroblast bioenergetic features was most effective in classifying the two forms of motor neurons disease, sALS and PLS, as indicated by the high area under the curve value (0.94) and the steep rise of true positive rate of the mean ROC curve (Fig 5b).

## Discussion

In this study, we characterized the bioenergetics of a large number of sALS and PLS primary skin fibroblast lines with the goal of finding disease classifiers. In addition, we studied the bioenergetics of a smaller cohort of fibroblast lines from the most common genetic form of fALS, *C9orf72,* to assess if bioenergetic features generalized to this form of the disease. We found that compared to healthy controls, sALS, PLS, and *C9orf72* shared higher mitochondrial membrane potential, and that sALS and PLS also shared features indicative of hypermetabolism, characterized by higher mitochondrial respiration and glycolytic fluxes.

So far, studies on metabolic function in sALS and fALS fibroblasts have been conducted in much smaller cohorts. In a study of 6 sALS and 10 control fibroblast lines, it was reported that sALS had a lower average OCR baseline [34]. However, the number of the samples was thirty-fold smaller than the one studied here. We think that the sample size is important in this case, because of the variability observed among individual lines in all bioenergetic assays. Importantly, when they looked selectively at the older (≥70 years of age at onset) patients, they found that ECAR was significantly higher than controls. This is in agreement with our results showing increased ECAR in sALS, PLS. Therefore, except for the OCR baseline result, which could differ because of the difference in sample sizes, glycolytic flux increase appears to be a common finding in the two studies. Furthermore, mitochondrial function measurements performed in 3 sALS fibroblast lines and 10 controls identified a defect of cytochrome c oxidase in sALS, which correlated with a lower respiratory activity, but only when cells were forced to respire with succinate as substrate after blocking complex I with rotenone [35]. In this study, we did not assess individual respiratory chain complexes, and since fibroblasts do not naturally utilize succinate as a major respiratory substrate, we did not analyze this pathway because of relatively low physiological significance. Instead, we performed measurements of respiration in intact cells allowed to utilize glucose and NADH-generating substrates, such as glutamine and pyruvate. Therefore, the differences in the findings may be attributable to the different sample size, but also to the different approaches utilized. Lastly, a recent study of 4 *C9orf72* and 4 control fibroblast lines found increased mitochondrial membrane potential in the *C9orf72* lines [36] similar to that found in our *C9orf72* cohort.

In our sALS and PLS fibroblast cohorts hypermetabolism was not accompanied by an increase in mitochondrial content or by a proportional increase in ATP content. Taken together, the data could be best interpreted as an adaptation to higher ATP demands, involving both oxidative and glycolytic pathways of energy generation. When cells were forced to maximize oxidative phosphorylation in galactose medium, the sALS and PLS were not capable of maintaining a faster respiratory rate than controls, suggesting that their capacity was close to maximal under glucose.

Further studies will be needed to dissect the mechanisms leading to hypermetabolism in ALS fibroblasts. However, we could hypothesize that several pathways may contribute to high ATP expenditure, including anabolic reactions, such as RNA and protein synthesis, catabolic reactions, such as protein degradation, vesicle acidification by V-ATPases, and ion homeostasis. The average values of several bioenergetic features were significantly different in PLS and sALS compared to controls, but also between sALS and PLS. The latter is a less aggressive form of motor neuron disease as compared to sALS, since it only affects the upper motor neurons and progresses more slowly. In light of these findings, it could be speculated that hypermetabolism could be a functional adaptation to increased ATP demands common to fibroblasts and neurons. Future studies utilizing neurons differentiated from fibroblast derived induced pluripotent cells or directly derived from fibroblasts will test the hypothesis that hypermetabolism is shared by fibroblasts and neurons.

We deemed that we could exploit the metabolic differences between disease and control lines and between sALS and PLS to identify disease classifiers. Despite the significant differences among groups in bioenergetic parameters, because of the large variability of the values, no single metric performed well enough to be used individually as a predictive classifier (e.g., to discriminate between controls, sALS and PLS). To overcome the constraints associated with single parameters, we opted to use multivariate analyses. We implemented a widely employed machine learning method of supervised multivariate analysis, the SVM, which takes all bioenergetics parameters from each cell line as input to make a prediction. This system generates its own hypothesis based on a learning process and produces a model for a decision boundary. The model was able to distinguish control and disease lines (sALS, PLS and *C9Orf72* combined) with moderate accuracy, likely because the variability among the disease groups. Importantly, we found that sALS and PLS fibroblasts could be distinguished with high specificity using the machine learning model.

This result suggests that a machine learning model based on fibroblast bioenergetics may serve as a classifier to predict, prior to a definite clinical diagnosis, whether a patient will develop sALS or the milder motor neuron disease PLS, a prediction that would have clear prognostic implications. Admittedly, machine learning works best with high number of examples and more diverse features than the ones currently available to us. Although our collection of ALS fibroblast lines is likely one of the largest in existence, we predict that expanding the database with additional lines and features will increase the performance of the classification model. In addition, since in this work we did not have enough *C9orf72* lines to be able to analyze them as a separate group, in the future it will be important increase the *C9orf72* cohort. This would allow us to include them in the multivariate analyses of disease groups, for example to predict whether a *C9orf72* patient will develop frontotemporal dementia, ALS, or both. Another development could include patients with different neuropathies, such as spinal muscular atrophy and hereditary spastic paraplegia, to assess whether the model’s power of distinction between sALS from PLS can be also extended to other forms of lower and upper motor neuron degeneration.

## Conclusions

We have identified bioenergetic markers of hypermetabolism in ALS fibroblasts. These findings will open new avenues of investigation of the molecular and biochemical mechanisms responsible for the bioenergetic modifications and their relationship to disease pathogenesis. We have also devised a novel approach that utilizes bioenergetic features to distinguish between fibroblast groups, which performed well in discriminating between sALS and PLS. Therefore, it is conceivable that analyses of fibroblasts bioenergetic features will help to stratify ALS patients into well-defined classes (e.g., hypermetabolic vs. normometabolic), to preselect patients entering clinical trials or to be used as post hoc criteria to interpret trial results. They could also help developing makers of prognosis and response to therapy, by implementing longitudinal studies on ALS fibroblast bioenergetics on subsequent skin biopsies obtained during disease progression and during the course of treatments.

## Additional file

Additional file 1: Figure S1.
*Flux analysis of control, sALS and PLS fibroblasts under forced oxidative metabolism in galactose medium*. Scatter plots of OCR baseline (A; Control mean: 3850.0, SD: 1936.7; sALS mean: 3307.5, SD: 1430.7; PLS mean: 2989.2, SD: 1164.7), oligomycin sensitive rate (B; Control mean: 3630.0, SD: 1772.0; sALS mean: 2835.0, SD: 1135.4; PLS mean: 2707.5, SD: 1138.9), maximal respiratory capacity (C; Control mean: 5325.8, SD: 2993.3; sALS mean: 3613.3, SD: 1662.5; PLS mean: 3741.7, SD: 2102.3), spare respiratory capacity (D; Control mean: 1478.8, SD: 1385.2; sALS mean: 306.8, SD: 589.2; PLS mean: 754.5, SD: 1120.1), ECAR baseline (E; Control mean: 1114.8, SD: 349.3; sALS mean: 1063.3, SD: 573.2; PLS mean: 1007.8, SD: 351.7), and OCR base/ECAR base (F; Control mean: 3.6, SD: 1.3; sALS mean: 3.5, SD: 1.4; PLS mean: 3.3, SD: 1.4). Values are shown comparing sALS, PLS, and control lines. Middle bars represent the average values and error bars show standard deviations. *p*-values are indicated where there was a significant difference between groups. n.s.: no significant difference. *n* = 12 sALS; n = 12 PLS, n = 12 controls. (DOCX 132 kb)

## Abbreviations

2DG: 2-Deoxy-D-glucose
AA + Rot: Antimycin A + Rotenone
ALS: Amyotrophic lateral sclerosis
ALSFRS: ALS Functional Rating Scale
BMI: Body mass index
DMEM: Dulbecco modified Eagle medium
ECAR: Extracellular acidification rate
FCCP: Cyanide p-trifluoromethoxyphenylhydrazone
FVC: Forced vital capacity
MTG: MitoTracker Green
OCR: Oxygen consumption rate
PBS: Phosphate buffered saline
PLS: Primary lateral sclerosis
ROC: Receiver operating characteristic
sALS: Sporadic ALS
SVM: Support vector machine
TMRM: Tetramethylrhodamine-methyl-ester
