# Supplementary material for: Fibroblast bioenergetics to classify amyotrophic lateral sclerosis patients
Source: Mol Neurodegener. 2017 Oct 24;12:76. doi: 10.1186/s13024-017-0217-5 (PMC5655870; doi:10.1186/s13024-017-0217-5)
Supplement: Additional file 1: Figure S1. — Flux analysis of control, sALS and PLS fibroblasts under forced oxidative metabolism in galactose medium. Scatter plots of OCR baseline (A; Control mean: 3850.0, SD: 1936.7; sALS mean: 3307.5, SD: 1430.7; PLS mean: 2989.2, SD: 1164.7), oligomycin sensitive rate (B; Control mean: 3630.0, SD: 1772.0; sALS mean: 2835.0, SD: 1135.4; PLS mean: 2707.5, SD: 1138.9), maximal respiratory capacity (C; Control mean: 5325.8, SD: 2993.3; sALS mean: 3613.3, SD: 1662.5; PLS mean: 3741.7, SD: 2102.3), spare respiratory capacity (D; Control mean: 1478.8, SD: 1385.2; sALS mean: 306.8, SD: 589.2; PLS mean: 754.5, SD: 1120.1), ECAR baseline (E; Control mean: 1114.8, SD: 349.3; sALS mean: 1063.3, SD: 573.2; PLS mean: 1007.8, SD: 351.7), and OCR base/ECAR base (F; Control mean: 3.6, SD: 1.3; sALS mean: 3.5, SD: 1.4; PLS mean: 3.3, SD: 1.4). Values are shown comparing sALS, PLS, and control lines. Middle bars represent the average values and error bars show standard deviations. p-values are indicated where there was a significant difference between groups. n.s.: no significant difference. n = 12 sALS; n = 12 PLS, n = 12 controls. (DOCX 132 kb) [file 13024_2017_217_MOESM1_ESM.docx]

**
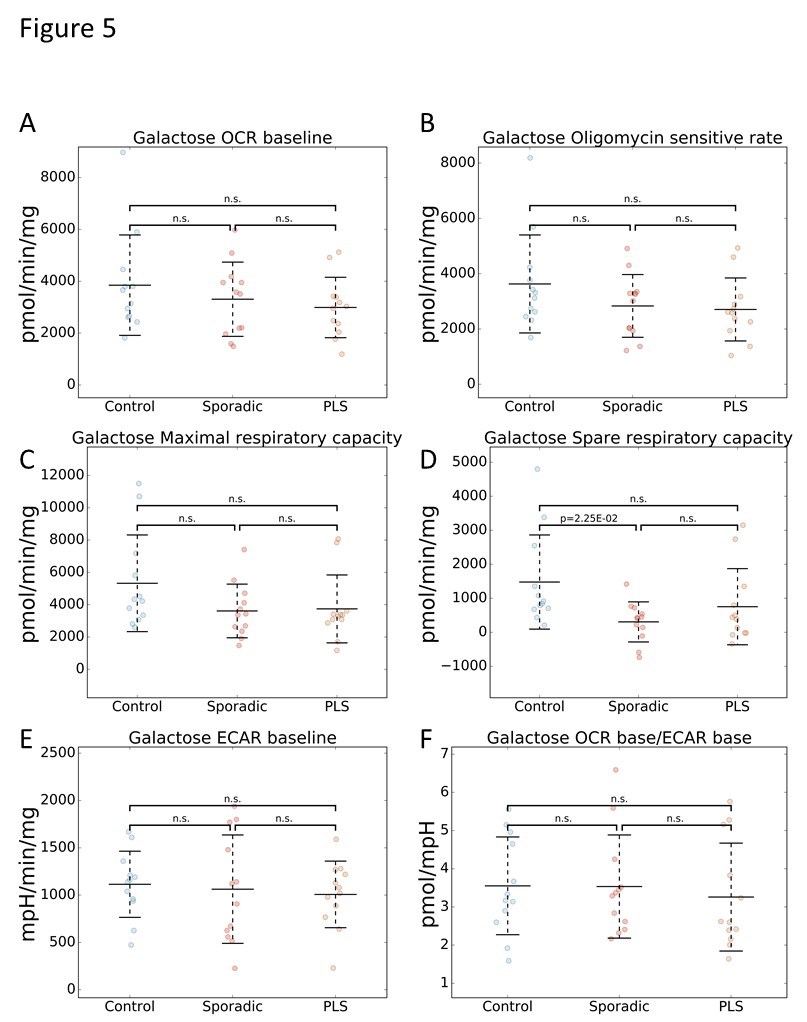
Konrad et al. Additional file 1**

**Figure S1**: *Flux analysis of control, sALS and PLS fibroblasts under forced oxidative metabolism in galactose medium*. Scatter plots of OCR baseline (A; Control mean: 3850.0, SD: 1936.7; sALS mean: 3307.5, SD: 1430.7; PLS mean: 2989.2, SD: 1164.7), oligomycin sensitive rate (B; Control mean: 3630.0, SD: 1772.0; sALS mean: 2835.0, SD: 1135.4; PLS mean: 2707.5, SD: 1138.9), maximal respiratory capacity (C; Control mean: 5325.8, SD: 2993.3; sALS mean: 3613.3, SD: 1662.5; PLS mean: 3741.7, SD: 2102.3), spare respiratory capacity (D; Control mean: 1478.8, SD: 1385.2; sALS mean: 306.8, SD: 589.2; PLS mean: 754.5, SD: 1120.1), ECAR baseline (E; Control mean: 1114.8, SD: 349.3; sALS mean: 1063.3, SD: 573.2; PLS mean: 1007.8, SD: 351.7), and OCR base/ECAR base (F; Control mean: 3.6, SD: 1.3; sALS mean: 3.5, SD: 1.4; PLS mean: 3.3, SD: 1.4). Values are shown comparing sALS, PLS, and control lines. Middle bars represent the average values and error bars show standard deviations. p-values are indicated where there was a significant difference between groups. n.s.: no significant difference. n=12 sALS; n=12 PLS, n=12 controls.
